# Food-cue affected motor response inhibition and self-reported dieting success: a pictorial affective shifting task

**DOI:** 10.3389/fpsyg.2014.00216

**Published:** 2014-03-13

**Authors:** Adrian Meule, Annika P. C. Lutz, Vera Krawietz, Judith Stützer, Claus Vögele, Andrea Kübler

**Affiliations:** ^1^Department of Psychology I, Institute of Psychology, University of WürzburgWürzburg, Germany; ^2^Research Group Self-regulation and Health, Research Unit INSIDE, Université du LuxembourgWalferdange, Luxembourg; ^3^Research Group on Health Psychology, University of LeuvenLeuven, Belgium

**Keywords:** food-cues, impulsivity, inhibitory control, response inhibition, go/no-go task, dieting success, body mass index

## Abstract

Behavioral inhibition is one of the basic facets of executive functioning and is closely related to self-regulation. Impulsive reactions, that is, low inhibitory control, have been associated with higher body mass index (BMI), binge eating, and other problem behaviors (e.g., substance abuse, pathological gambling, etc.). Nevertheless, studies which investigated the direct influence of food-cues on behavioral inhibition have been fairly inconsistent. In the current studies, we investigated food-cue affected behavioral inhibition in young women. For this purpose, we used a go/no-go task with pictorial food and neutral stimuli in which stimulus-response mapping is reversed after every other block (*affective shifting task*). In study 1, hungry participants showed faster reaction times to and omitted fewer food than neutral targets. Low dieting success and higher BMI were associated with behavioral disinhibition in food relative to neutral blocks. In study 2, both hungry and satiated individuals were investigated. Satiation did not influence overall task performance, but modulated associations of task performance with dieting success and self-reported impulsivity. When satiated, increased food craving during the task was associated with low dieting success, possibly indicating a preload-disinhibition effect following food intake. Food-cues elicited automatic action and approach tendencies regardless of dieting success, self-reported impulsivity, or current hunger levels. Yet, associations between dieting success, impulsivity, and behavioral food-cue responses were modulated by hunger and satiation. Future research investigating clinical samples and including other salient non-food stimuli as control category is warranted.

## Introduction

In western or westernized countries, highly palatable and high caloric food is omnipresent and easily accessible. Therefore, constant self-regulation over automatic action tendencies to consume these foods is inevitable to prevent weight gain (Lowe, 2003; Cohen and Farley, 2008). An important prerequisite for successful self-regulation are executive functions (Hofmann et al., 2012). Recent research has focused on three basic facets of executive functions: (1) working memory, (2) inhibition, and (3) cognitive flexibility (Hofmann et al., 2012; Diamond, 2013). Working memory refers to the maintenance and updating of relevant information, inhibition to withhold pre-potent impulses and cognitive flexibility involves mental set shifting, for example, changing perspectives or approaches to a problem and flexibly adjusting to new demands, rules or priorities (Hofmann et al., 2012; Diamond, 2013). Emerging evidence suggests that those executive functions support important mechanisms in achieving self-regulatory goals and that temporary reductions in executive functioning may be a common mechanism contributing to self-regulatory failure (Hofmann et al., 2012).

In the field of eating behavior, it has been shown that food-cues and food craving consume self-regulatory resources and diminish working memory performance (Kemps and Tiggemann, 2010; Meule et al., 2012e). Consequently, higher working memory capacity may contribute to successful eating-related self-regulation (Hofmann et al., 2012). With regard to cognitive flexibility, little work has addressed its relation to self-regulation (Hofmann et al., 2012). For instance, it was suggested that flexible dieting behavior is associated with better eating-related self-regulation as compared to rigid dieting (Meule et al., 2011c; Westenhoefer et al., 2013). Recently, Delgado-Rico et al. (2012) found that higher body mass index (BMI) was associated with cognitive rigidity as measured with a Stroop response switching task.

### Behavioral inhibition and eating behavior

In contrast to working memory and cognitive flexibility, there are plenty of studies investigating the link between behavioral inhibition and eating regulation. Two of the most often used tasks for measuring behavioral inhibition are go/no-go tasks and the stop-signal paradigm. Go/no-go tasks involve the instruction to respond to a certain stimulus (e.g., by pressing a button), but to inhibit this response to another stimulus. In stop-signal tasks, the go signal is presented on every trial, but in a minority of trials a stop-signal is presented shortly after onset of the go signal indicating that one should not press the button on that trial. Stop-signal delay is adjusted dynamically and a stop-signal reaction time is calculated with higher values indicating lower inhibitory performance (Logan et al., 1997). In the following, we will use the terms behavioral inhibition, response inhibition, and inhibitory control interchangeably in the sense of reflecting number of commission errors in go/no-go tasks or stop-signal reaction time in stop-signal tasks^1^. Additionally, other task performance indices can be calculated in behavioral inhibition tasks, for example, reaction times in correct go-trials and omission errors. While omission errors probably reflect lapses of attention, interpretation of reaction times is not straightforward as they neither represent a pure measure of behavioral inhibition nor distinct attentional mechanisms (Schulz et al., 2007).

A number of studies have shown that diminished inhibitory performance is associated with overeating (see Guerrieri et al., 2008 for a review). Lower behavioral inhibition was demonstrated in patients with bulimia nervosa (Rosval et al., 2006; Wu et al., 2013), restrained eaters (Nederkoorn et al., 2004), and obese children (Nederkoorn et al., 2006a, 2007) and adults (Nederkoorn et al., 2006b), as compared to controls. Furthermore, response inhibition has been found to moderate food consumption such that particularly those restrained eaters that exhibited low inhibition ate more in a laboratory setting (Jansen et al., 2009; Meule et al., 2011a). Hence, it can be concluded that lower behavioral inhibition is associated with lower eating-related self-regulation, as operationalized by higher laboratory food intake, higher BMI, or binge eating. These findings, however, could not be confirmed by a number of studies failing to show decreased go/no-go or stop-signal task performance in patients with bulimia, binge eating disorder or obesity compared with controls (Claes et al., 2006, 2012; Galimberti et al., 2012; Hendrick et al., 2012; Van den Eynde et al., 2012; Wu et al., 2013).

In his *hedonic-inhibitory model*, Appelhans (2009) integrated the appetitive motivation in relation to food (what he termed *hedonic feeding*) and the inhibition of this appetitive motivation. In accordance with the assumption that those two mechanisms interact in the control of food intake, Appelhans et al. (2011) could show that an interactive effect of high food reward sensitivity (as measured with the *Power of Food Scale*) and low inhibitory control (as measured with a delay discounting task) predicted laboratory food intake of palatable food items in overweight and obese women. Similar interactive effects were found by others such that low inhibitory control (as measured with the stop-signal paradigm) in combination with a high implicit preference for snack foods predicted laboratory candy consumption (Hofmann et al., 2009) and 1-year weight gain (Nederkoorn et al., 2010). As all of these studies assessed a general capacity for inhibitory control, Appelhans and colleagues came to the conclusion that “[…] there is clearly a need to develop tasks which specifically measure inhibitory control in the context of food rewards” (Appelhans et al., 2011, p. 2180). This opinion has been echoed by other researchers, for example in the context of neuroimaging research in an attempt “[…] to break down the mechanisms underlying excessive food intake by moving beyond food intake and food cue paradigms into the realm of cognitive tasks designed to tap impulsive behavior such as the go/no-go task […]” (Carnell et al., 2012, p. 54).

### Behavioral inhibition and food-cues

In addition to those studies, which relate performance in non-food related motor response inhibition tasks to eating behavior, there are some studies, which extend those tasks with concurrent presentation of food-cues (Table 1). Thus, these tasks measure reactive behavioral inhibition, which refers to a bottom-up interruption of ongoing behavior due to motivation conditions attached to the task (Nigg, 2000; Schulz et al., 2007; Claes et al., 2012). According to Appelhans' framework (Appelhans, 2009; Appelhans et al., 2011) one would expect that individuals who are prone to overeating, for example, unsuccessful dieters or individuals with obesity or binge eating behaviors, should exhibit disinhibition specifically in response to food-cues in motor response inhibition tasks due to their low inhibitory control and high food reward sensitivity. However, studies which investigated food-cue affected behavioral inhibition are fairly inconsistent. That is, some studies did not reveal an influence of food-cues on behavioral inhibition, some found an influence across all participants, and some only found an influence in a specific subgroup of participants (Table 1).

**Table 1:** **Studies which investigated the influence of food-cues on behavioral inhibition**.

**Study**	**Participants**	***N***	**Task**	**Stimuli**	**Effect of food-cues on inhibition**	**Main results**
Batterink et al., 2010	Normal- and overweight adolescent girls	35	Go/no-go	Pictures of vegetables in go-trials, pictures of desserts in no-go-trials	n.a.	BMI negatively correlated with reaction times and positively correlated with commission errors
					(no control condition)	
Houben, 2011	Normal- and overweight women	29	SST	Pictures of food and neutral objects; One type of food either always paired with an auditory stop-signal, never paired with the stop-signal or paired with the stop-signal in half of the trials	n.a.	Decreased consumption of the stop food in participants with low inhibitory control abilities
					(not analyzed)	
Houben and Jansen, 2011	Normal- and overweight women	63	Go/no-go	Letters in one of four corners of pictures of food and neutral objects; Pictures of chocolate either always paired with go-cues, always paired with stop-cues or paired with half of the go-cues and half of the stop-cues	n.a.	Participants in the chocolate/no-go condition consumed less chocolate compared with the control condition
					(not analyzed)	
Houben et al., 2012b	Normal- and overweight women	50	SST	General SST with letters (X and O) and food-specific SST (four pictures of food presented either in landscape or portrait format)	yes	Unsuccessful weight regulators had higher SSRT after food exposure compared with the control condition in the food-specific SST; this effect was not present in the general SST
Jasinska et al., 2012	Underweight-to-obese men and women	204	Go/no-go	Letters flanked by two identical pictures of food	n.a.	Commission errors positively associated with emotional eating scores and negatively with healthy food choices
					(no control condition)	
Loeber et al., 2012	Normal-weight and obese adults	40	Go/no-go	Food and object words	Yes	All participants responded faster in go-trials with food words compared with object words and made more commission errors in response to food words (i.e., when neutral words were targets) compared with neutral words (i.e., when food words were targets)
Loeber et al., 2013	Normal-weight adults	48	Go/no-go	Food and clothing words	Yes	All participants made more commission errors in response to food words (i.e., when neutral words were targets) compared with neutral words (i.e., when food words were targets). This effect was particularly pronounced in participants with high self-reported hunger, but was unrelated to blood glucose levels
Meule et al., 2011a	Normal-weight women	61	Go/no-go	Letters encircled by pictures of food or objects	None	Restrained eaters responded slower than unrestrained eaters in food blocks and made less commission errors in all blocks compared with unrestrained eaters
Meule et al., 2012c	Normal-weight women	50	Go/no-go	Letters superimposed over pictures of food or objects	None	Women with food addiction symptoms responded faster in food blocks compared with neutral blocks
Meule et al., 2014	Normal-weight women	50	SST	Food and neutral pictures	Yes	Higher SSRT in response to food pictures was related to higher food craving after the task
Mobbs et al., 2008	Normal-weight women with bulimia nervosa and controls	36	Go/no-go	Food and object words	Yes?	All participants responded faster to and showed better discrimination of food targets than neutral targets; bulimic patients show lower inhibition in the food part of the task (i.e., in both food and neutral blocks) compared with controls and reacted faster overall
Mobbs et al., 2011	Obese adults with and without binge eating disorder and normal-weight controls	48	Go/no-go	Food and object words	Yes	All participants responded faster to food targets than neutral targets; obese BED slower than obese non-BED in detecting neutral targets in the shift condition; obese participants generally made more errors than controls; all participants made more commission errors in response to neutral words (i.e., when food words were targets) compared with food words (i.e., when neutral words were targets); obese BED generally more commission and omission errors than obese without BED; all participants had a positive bias for food words relative to control words
Nederkoorn et al., 2004	Normal-weight women	63	SST	Letters; food exposure before and during second half of the experiment	None	Restrained eaters had higher SSRT compared with unrestrained eaters
Nederkoorn et al., 2012	Lean and overweight children	91	SST	Pictures of food or toy on the left or right side of the screen; incentive (earn candy or toy points) for correct and fast responses	Yes	Children had higher SSRT in response to food pictures as compared with toy pictures; this effect was even more pronounced in overweight children
Veling et al., 2011; Study 1	Normal-weight women	38	Go/no-go	Letters superimposed over pictures of food or objects	n.a.	Dieters responded slower in subsequent action probe trials when foods had been paired with no-go cues in the inhibition induction phase
					(not analyzed)	

*n.a., not available; BMI, body mass index; SST, stop-signal task; SSRT, stop-signal reaction time; BED, binge eating disorder*.

Firstly, it has to be noted that studies differ substantially in methodology, for example, in the type of samples studied (differing in psychopathology, body mass, age, or gender), the type of task used (go/no-go vs. stop-signal) or study design and stimuli presentation (words, pictures, real food exposure, position of stimuli on the screen, etc.). Secondly, in some studies indices of behavioral inhibition in response to food-cues were not analyzed (or not reported) because the task was only used as a training or inhibition induction phase (Houben, 2011; Houben and Jansen, 2011; Veling et al., 2011). Thirdly, one study found a positive relationship between number of commission errors in response to pictures of desserts and BMI (Batterink et al., 2010) and, in another study, the number of commission errors was positively associated with emotional eating scores and negatively associated with healthy food choices (Jasinska et al., 2012). However, neither of those studies had a control condition which means that it remains unclear if behavioral inhibition was actually influenced by the food stimuli or if the same results could have been obtained with a neutral go/no-go task. Fourthly, some studies found an influence of food-cues on reaction times or group differences in general behavioral inhibition, but no specific influence of food-cues (or food exposure) on behavioral inhibition (Nederkoorn et al., 2004; Meule et al., 2011a, 2012c). Finally, in some studies an influence of food-cues on behavioral inhibition could be observed (Table 1). Using a neutral and a food-specific stop-signal task, Houben et al. (2012b) found that unsuccessful dieters were less behaviorally inhibited in response to food-cues after food exposure as compared to a control condition and this effect could not be found in successful dieters or in the neutral stop-signal task. In another stop-signal task, Nederkoorn et al. (2012) found that particularly overweight children exhibited lower response inhibition in response to food pictures as compared with toy pictures. Most recently, higher SSRT in response to food pictures was related to higher food craving after the task in young women (Meule et al., 2014).

### Food-related affective shifting tasks

Four studies used a lexical go/no-go task in which participants were required to respond to either food or neutral words, but to inhibit reactions to the other category. Stimulus-response mapping is reversed after every other block and, therefore, this task measures both behavioral inhibition and cognitive flexibility (*affective shifting task*, AST; Mobbs et al., 2008, 2011). Decreased behavioral inhibition in this task is indexed by an increasing number of commission errors. Cognitive flexibility can be assessed by general differences in task performance between *shift* and *non-shift* blocks (see description of the AST below).

In a first study, all participants responded faster to food than neutral targets (Mobbs et al., 2008). Furthermore, patients with bulimia nervosa exhibited lower response inhibition (in both food and neutral blocks) compared to controls, but not in a body-related AST (Mobbs et al., 2008). In a second study, again all participants responded faster to food targets relative to neutral targets (Mobbs et al., 2011). Obese participants generally committed more errors than controls (Mobbs et al., 2011). Importantly, all participants made more commission errors in response to neutral words (i.e., when food words were targets) as compared to food words (i.e., when neutral words were targets) (Mobbs et al., 2011). In a third study, it was confirmed that all participants responded faster to food words relative to object words (Loeber et al., 2012). However, in this study participants made more commission errors in response to food words (i.e., when neutral words were targets) as compared to neutral words (i.e., when food words were targets) (Loeber et al., 2012). No differences were found between normal-weight and obese participants (Loeber et al., 2012). Most recently, it could be replicated in normal-weight individuals that the number of commission errors was higher in response to food than neutral words (Loeber et al., 2013). This effect was particularly pronounced in participants with high subjectively rated hunger, but was unrelated to blood glucose levels (Loeber et al., 2013).

To summarize, studies using food-related ASTs indeed found an influence of food-cues on behavioral inhibition. Yet, several issues remain inconclusive. Firstly, although differences in task performance were found between food and neutral targets, contrary to expectations, this effect was unrelated to body mass or trait eating behaviors. As a result, alternative explanations such as simple category size effect (see Discussion) cannot be excluded. Secondly, studies are contradictory as it is not clear if behavioral disinhibition can be specifically observed in response to food words (although participants are instructed to inhibit those reactions) (Loeber et al., 2012, 2013) or in response to neutral distractors (when participants are required to respond to food words) (Mobbs et al., 2011). Thirdly, it is unclear how food-related response inhibition is related to current nutritional status as it was associated with self-reported hunger, but not blood glucose levels (Loeber et al., 2013).

In the current studies, we aimed at clarifying and extending those previous findings. For this purpose, we used a food-related AST similar to the studies described above (Mobbs et al., 2008, 2011; Loeber et al., 2012, 2013) but used pictures of food and neutral objects as food pictures have been found to produce stronger effects as compared to food words in cognitive tasks (Brooks et al., 2011). Based on previous findings, we expected that participants would respond faster to food targets than neutral targets. In addition, we expected that there would be differences in behavioral inhibition between stimulus types which may be seen in higher disinhibition (i.e., more commission errors) in food blocks (Mobbs et al., 2011) or in neutral blocks (Loeber et al., 2012). Finally, we explored if task performance was associated with food deprivation, current food craving, BMI, self-reported dieting success and impulsivity with correlational and regression analyses.

## Study 1

### Materials and methods

#### Participants

Female participants were recruited among students at the University of Würzburg, Germany. Advertisements were posted on campus and using a mailing list of a student council. Women who responded to the advertisements were contacted by phone (*N* = 82) and screened for exclusion criteria which included mental disorders, psychoactive medication, under- or overweight (BMI < 17.5 or > 25 kg/m^2^), and age > 40 years. Only women with normal-weight were included because only few participants of the screened sample were in the overweight range and, therefore, BMI distribution would have been skewed. For the same reason, only women younger than 40 years were considered eligible. A total of *n* = 50 participants took part in the study. Mean age was *M* = 22.32 years (*SD* = 3.03) and mean BMI *M* = 21.45 kg/m^2^ (*SD* = 2.67). Eighteen participants indicated that they were currently trying to control their weight (i.e., dieters). Five participants were smokers. Food deprivation (i.e., mean time since the last meal) was *M* = 5.20 h (*SD* = 2.81). Participants either received course credits or €20 for participation^2^.

#### Questionnaires

***Perceived Self-Regulatory Success in Dieting Scale (PSRS)***. The PSRS (Fishbach et al., 2003) was used to assess dieting success. In this three-item questionnaire, participants have to rate on 7-point scales how successful they are in watching their weight, in losing weight, and how difficult it is for them to stay in shape. Validity of the PSRS has been shown by negative associations with BMI, rigid dieting strategies and other measures of disinhibited eating while it is positively related to flexible dieting strategies (Meule et al., 2011c, 2012a,d). Internal consistency of the German version is α > 0.70 (Meule et al., 2012d) and was α = 0.79 in the current study.

***Barratt Impulsiveness Scale—Short Version (BIS-15)***. The BIS-15 was proposed by Spinella (2007) as short version of the BIS-11 (Patton et al., 1995) for the measurement of impulsivity on the dimensions *motor, attentional*, and *non*-*planning impulsivity*. Instead of 30 items as in the long version, it consists of 15 items only. Convergent validity of the BIS-15 has been shown by moderate to strong relationships with the *Frontal Systems Behavior Scale* and the *UPPS Impulsive Behavior Scale* while discriminant validity has been indicated by weak correlations with sensation seeking (Spinella, 2007; Meule et al., 2011b). Internal consistency of the German version is α = 0.81 (Meule et al., 2011b). Only the total score was used in the current study and internal consistency was α = 0.79.

***Food Cravings Questionnaire—State (FCQ-S)***. The FCQ-S (Cepeda-Benito et al., 2000) was used to measure current food craving. This 15-item questionnaire assesses momentary food craving on the dimensions intense desire to eat, anticipation of positive reinforcement that may result from eating, anticipation of relief from negative states and feelings as a result of eating, lack of control over eating, and craving as a physiological state (Cepeda-Benito et al., 2000). Validity of the FCQ-S has been indicated by positive associations with length of food deprivation and current negative affect (Cepeda-Benito et al., 2003; Meule et al., 2012a). Moreover, the FCQ-S has been found to be sensitive to meal consumption and food-cue exposure such that state cravings decreased after breakfast (Cepeda-Benito et al., 2000; Vander Wal et al., 2007) and increased after performing a cognitive task involving food pictures (Meule et al., 2012e). Subscales are highly inter-correlated and internal consistency of the total score is α = 0.92 (Meule et al., 2012a). Therefore, we only used the total score for our analyses and internal consistency was α = 0.90 in the current study.

#### Affective shifting task (AST)

**Figure 1:**
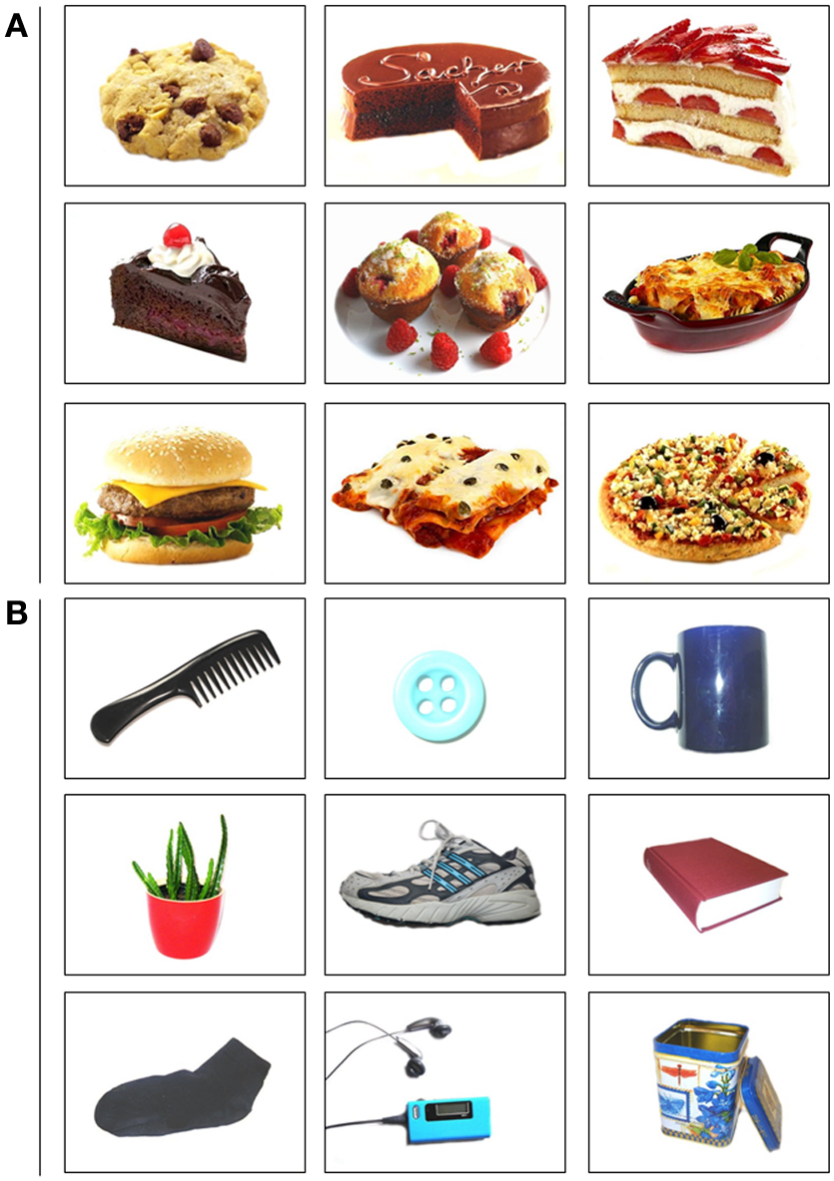
**Stimulus set of (A) food pictures and (B) neutral pictures in study 1**.

The AST is a go/no-go task which has been previously employed using emotional (Murphy et al., 1999), alcohol-related (Noël et al., 2005, 2007; Adams et al., 2013) and food- or body-related (Mobbs et al., 2008, 2011; Loeber et al., 2012, 2013) words. In the current study, we used a modification of this task with pictures of food and neutral objects. Food items were pictures of high caloric, palatable sweet and savory foods. Neutral pictures were common household items. All pictures were edited to be homogeneous with respect to background color (Figure 1). The program was compiled using E-prime 2.0 (Psychology Software Tools Inc., Pittsburgh, PA) and displayed on a LCD TFT 22” screen. Participants were instructed to press a response button as quickly as possible when a target was presented but withhold responses to distractors. The task was separated into 16 blocks consisting of 18 trials each (= 288 trials in total). Within each block, every picture was shown once, that is, half of the pictures were targets and half were distractors. Pictures were presented one by one for 500 ms in a randomized order. A blank screen was presented during inter-trial interval for 900 ms or participants received a feedback in case of a false reaction or omission. Before each block, either food (F) or objects (O) was specified as target category. The order of blocks was either FFOOFFOOFFOOFFOO or OOFFOOFFOOFFOOFF (counterbalanced across subjects). Due to this arrangement, four blocks of each target category were *shift* blocks in which participants had to reverse stimulus-response associations of the previous block, and four blocks were *non-shift* blocks in which stimulus-response associations were the same as in the previous block. To ensure that the first block could be analyzed as a shift block, a practice block using the opposite target category was run prior to the test blocks. The whole task lasted for approximately 10 min.

#### Procedure

Participants were tested between 9:00 a.m. and 6:00 p.m. (median of testing time was 12:00 noon). All participants were asked not to consume food, caffeine, nicotine, or alcohol at least 3 h before the experiment. After participants had performed the AST, they immediately filled out the FCQ-S and reported the hours that had elapsed since their last meal. Completion of the other questionnaires and measurement of participants' height and weight was conducted either on the same day or within 1–2 weeks after the experiment, depending on individual assignment to experimental conditions.

#### Data analysis

Trials with a reaction time of less than 150 ms, reflecting anticipation, were excluded from analyses. Measures of interest were reaction times (ms) in go-trials (i.e., time taken to respond to each target), number of commission errors (i.e., responses to distractors), and omission errors (i.e., failure to respond to targets). Correlations between the different task performance indices are shown in Table 2.

**Table 2:** **Pearson correlation coefficients between indices of task performance**.

	**Study 1 (***N*** = **50**)**			**Study 2 (***N*** = **102**)**		
	**1**	**2**	**3**	**1**	**2**	**3**
1. Reaction times	–	−0.03	0.70^***^	–	−0.08	0.66^***^
2. Commission errors	−0.03	–	0.03	−0.08	–	0.33^**^
3. Omission errors	0.70^***^	0.03	–	0.66^***^	0.33^**^	–

** p < 0.01;

*** *p < 0.001*.

A 2 (*target type*: food vs. object) × 2 (*block type*: shift vs. non-shift) ANOVA for repeated measures was calculated for each dependent variable (reaction time, omission, and commission errors). *Post-hoc t*-tests were calculated in case of significant interactions. In addition, we calculated correlations between task performance parameters in food blocks, neutral blocks and difference scores (food minus neutral) with BMI, food deprivation, and questionnaire measures. Results were considered as significant at an α level of *p* = 0.05. Results marked as *ns* refer to *p*-values > 0.05.

## Results

### Task performance

#### Reaction times

**Figure 2:**
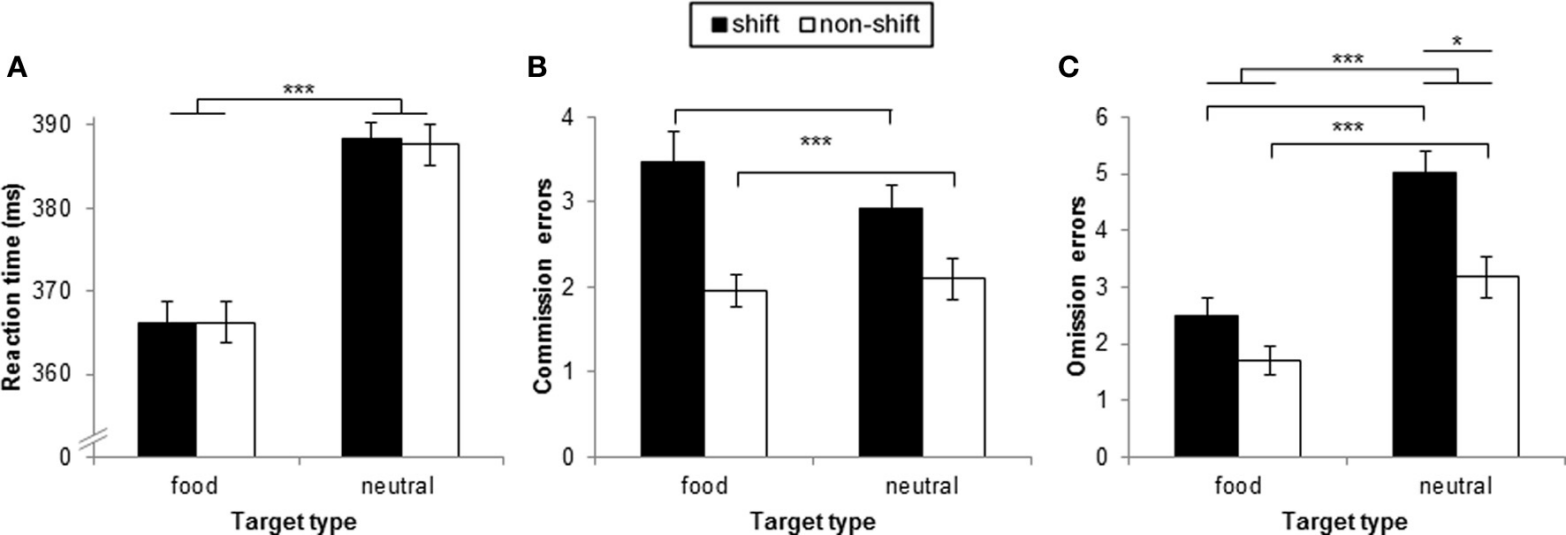
**Task performance as a function of target type and block type in study 1**. Displayed is **(A)** reaction time in go-trials in ms, **(B)** number of commission, and **(C)** omission errors. Error bars indicate standard errors of the mean. ^***^p < 0.001, ^*^p < 0.05.

There was a significant main effect for target type [*F*_(1, 49)_ = 202.11, *p* < 0.001, η^2^_*p*_ = 0.81] indicating faster reaction times in response to food targets (*M* = 366.15 ms, *SD* = 16.60) than to neutral targets (*M* = 387.98 ms, *SD* = 14.70). Neither the main effect for block type [*F*_(1, 49)_ = 0.05, *ns*, η^2^_*p*_ = 0.00] nor the interaction target type × block type [*F*_(1, 49)_ = 0.81, *ns*, η^2^_*p*_ = 0.00] were significant (Figure 2A).

#### Commission errors

The main effect for target type was not significant [*F*_(1, 49)_ = 0.54, *ns*, η^2^_*p*_ = 0.01], but there was a significant main effect for block type [*F*_(1, 49)_ = 23.82, *p* < 0.001, η^2^_*p*_ = 0.33] indicating more commission errors in shift blocks (*M* = 3.2, *SD* = 1.75) than in non-shift blocks (*M* = 2.03, *SD* = 1.22). The interaction target type × block type was not significant [*F*_(1, 49)_ = 3.84, *ns*, η^2^_*p*_ = 0.07, Figure 2B].

#### Omission errors

There were significant main effects for target type [*F*_(1, 49)_ = 56.65, *p* < 0.001, η^2^_*p*_ = 0.54] and block type [*F*_(1, 49)_ = 33.65, *p* < 0.001, η^2^_*p*_ = 0.41] indicating more omission errors in response to neutral targets (*M* = 4.10, *SD* = 2.18) than to food targets (*M* = 2.1, *SD* = 1.79) and more omission errors in shift blocks (*M* = 3.76, *SD* = 2.11) than in non-shift blocks (*M* = 2.44, *SD* = 1.73). These main effects were further qualified by a significant interaction target type × block type [*F*_(1, 49)_ = 4.21, *p* < 0.05, η^2^_*p*_ = 0.08]. *Post-hoc t*-test indicated that the difference between shift and non-shift blocks was particularly pronounced for neutral targets [*M*_(shift−non−shift)_ = 1.84, *SD* = 3.01] as compared to food targets [*M*_(shift−non−shift)_ = 0.80, *SD* = 1.60, *t*_(49)_ = 2.05, *p* < 0.05, Figure 2C].

### Correlational analyses

BMI was negatively correlated with dieting success (*r* = −0.67, *p* < 0.001). BMI was positively and dieting success negatively correlated with difference scores for commission errors (Table 3). With higher BMI and lower dieting success, participants committed more errors in food blocks as compared to neutral blocks. Task performance was unrelated to food deprivation, current food craving, and impulsivity (Table 3).

**Table 3:** **Pearson correlation coefficients between task performance and BMI, food deprivation, and questionnaire measures (study 1)**.

	**Reaction times**			**Commission errors**			**Omission errors**		
	**Food**	**Neutral**	**Food-neutral**	**Food**	**Neutral**	**Food-neutral**	**Food**	**Neutral**	**Food-neutral**
Food deprivation	0.17	0.07	0.17	−0.19	−0.00	−0.15	0.02	0.04	−0.02
FCQ-S	0.13	0.26	−0.15	0.18	0.09	0.08	0.17	0.27	−0.17
BMI	0.19	0.12	0.12	0.26	−0.17	0.35^*^	0.08	−0.02	0.09
PSRS	−0.01	−0.01	−0.00	−0.10	0.27	−0.29^*^	0.11	0.15	−0.07
BIS-15	−0.14	−0.04	−0.16	0.06	0.02	0.03	0.07	−0.05	0.12

*FCQ-S, Food Cravings Questionnaire—State; BMI, Body mass index; PSRS, Perceived Self-Regulatory Success in Dieting Scale; BIS-15, Barratt Impulsiveness Scale—Short form*.

* *p < 0.05*.

## Conclusion of study 1

Study 1 showed that participants reacted faster in response to food than neutral targets. They also omitted fewer food than neutral targets. Commission errors did not differ between food and neutral blocks. Thus, these results suggest that, although differences in task performance between food and neutral cues can be found, food-cues do not affect behavioral inhibition. However, participants only committed few errors overall (*M* = 5.44 errors in food blocks and *M* = 5.02 errors in neutral blocks) and, thus, the lack of a difference in behavioral inhibition may be due to a ceiling effect.

Higher BMI and lower dieting success were associated with higher disinhibition (i.e., more commission errors) in food relative to neutral blocks. Food deprivation, state food craving after the task, and impulsivity were unrelated to task performance. Thus, rather unsuccessful control over food intake appears to be related to impaired behavioral inhibition when confronted with palatable food-cues and this association seems to be independent of current food deprivation, food craving, or trait impulsivity.

## Study 2

In study 2, the very same state and trait measures were used [i.e., food deprivation (time since last meal), state food craving, BMI, dieting success, and impulsivity] and, again, a pictorial version of the AST was administered; yet, the following modifications in study design were made:

1. As participants only committed few errors in study 1, the number of trials was increased from 288 to 320 trials in order to produce a higher number of commission errors.
2. In study 1, state food craving *after* the task was neither related to task performance nor to BMI or any self-report measure. Yet, state food craving usually increases during such tasks (cf. Meule et al., 2012e; Meule and Kübler, in revision) and, consequently, it is possible that craving *before* or increases *during* the task could be related to task performance, BMI, or self-report measures. Thus, in study 2, state food craving was also assessed before the task in addition to its assessment after the task.
3. Differences in task performance, that is, reaction times and omission errors, between food and neutral stimuli in study 1 can be due to a number of reasons, for example, stimuli characteristics (e.g., visual complexity, category size) or hunger (e.g., that participants react faster to food stimuli when they are hungry). Thus, in study 2, food and neutral pictures were matched with regard to physical features such as visual complexity. In addition, nutritional status was manipulated such that half of participants consumed food in the laboratory (satiated group, *n* = 51) and the other half was food deprived for several hours (hungry group, *n* = 51).

The following hypotheses were formulated:

1. *Current food craving*: Food consumption would reduce self-reported food craving in the satiated group and, thus, the satiated group would report lower food craving as indicated by scores on the FCQ-S before and after the task as compared to the hungry group. Yet, self-reported food craving was expected to be higher after the task as compared to before in both groups as this can usually be found when cognitive tasks with pictorial, palatable food stimuli are investigated (Meule et al., 2012e; Meule and Kübler, in revision).
2. *Correlates of current food craving*: Although we expected a general increase in state food craving during the AST because of food-cue exposure, there are also inter-individual differences in food-cue reactivity. For example, higher food-cue reactivity has been observed in individuals with higher BMI (Jansen et al., 2003), restrained eating (Fedoroff et al., 1997), and higher self-reported impulsivity (Tetley et al., 2010). Furthermore, food-cue reactivity in rather unsuccessful dieters is increased after ingestion of a high-calorie preload (Stroebe, 2008). Thus, we explored if changes in state food craving during the AST were associated with current food deprivation or trait variables (BMI, dieting success, impulsivity) and if those associations were modulated by food intake.
3. *Task performance*: We expected to replicate findings of study 1 that participants would react faster to and omit fewer food than neutral targets. As we increased the number of trials, we expected that participants would commit more errors overall and, subsequently, that we now also would find differences in commission errors between food and neutral blocks. As noted above, existing literature yielded opposing results, so we formulated a non-directional hypothesis that participants may commit more errors in food blocks (Mobbs et al., 2011) or in neutral blocks (Loeber et al., 2012). Moreover, it was found recently that differences in commission and omission errors between blocks with lexical food and neutral stimuli could particularly be found in individuals with high self-reported hunger (Loeber et al., 2013). Thus, we expected that, in the satiated group, differences in task performance between food and neutral blocks would be attenuated.
4. *Correlates of task performance*: Based on the findings of study 1, we expected that higher BMI and lower dieting success would be associated with more commission errors in food relative to neutral blocks. As participants were expected to commit more errors overall, we now hypothesized that the number of commission errors would be positively associated with self-reported impulsivity (Aichert et al., 2012). All analyses were conducted using commission errors as well as reaction time and omission errors in order to elucidate if results were specifically related to behavioral inhibition or to overall task performance. Finally, we explored if task performance would be related to state variables, that is, food deprivation and current food craving, and if possible associations between state and trait variables with task performance would be modulated by group (i.e., hunger and satiation).

### Materials and methods

#### Participants

Female participants were recruited among students at the University of Würzburg, Germany. A total of *N* = 102 participants took part in the study. Similar to study 1, all participants were younger than 40 years (*M* = 22.76 years, *SD* = 3.72). However, no restrictions were imposed regarding BMI. As a result, mean BMI was slightly higher, but still comparable to study 1 (*M* = 22.11 kg/m^2^, *SD* = 3.36). Thirty-one participants (30.4%) indicated that they were currently trying to control their weight (i.e., were dieters). Eight participants (7.8%) were smokers. Food deprivation (i.e., mean time since the last meal) was *M* = 7.5 h (*SD* = 4.93). Participants either received course credits or €6 for participation.

#### Questionnaires

The same questionnaires as in study 1 were used.

#### AST

**Figure 3:**
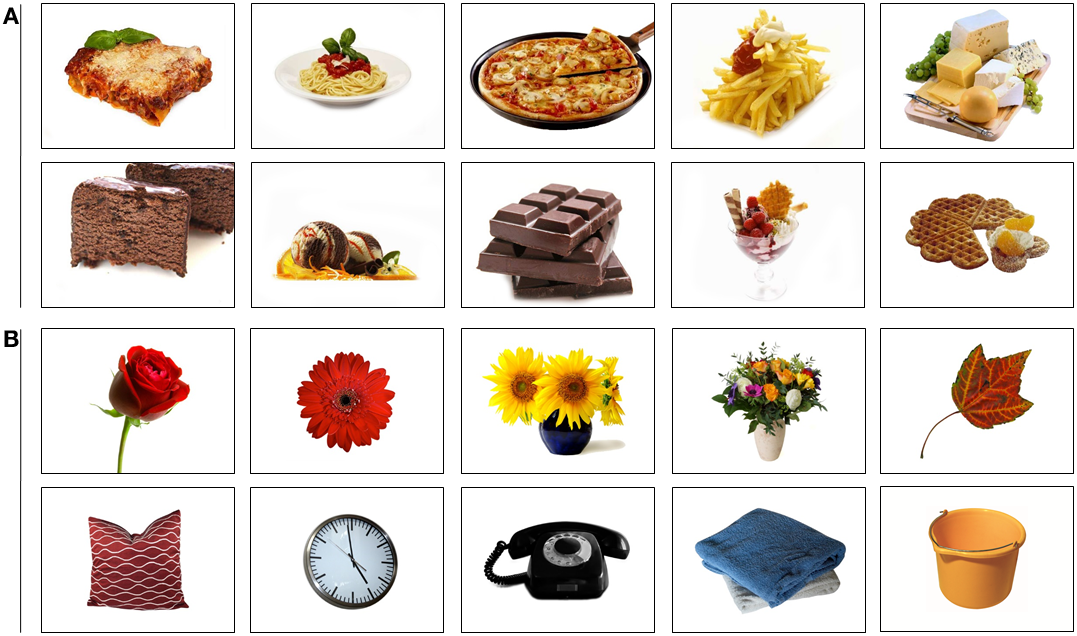
**Stimulus set of (A) food pictures and (B) neutral pictures in study 2**.

Again, a pictorial version of the AST task was used. Ten pictures of food items and 10 neutral pictures (Figure 3) were selected from the *food.pics* database, which includes information on physical features, among others (see www.food-pics.sbg.ac.at; Meule and Blechert, 2012)^3^. Food and neutral stimuli did not differ in visual complexity (jpg compression, edge detection, subjective ratings) and RGB brightness and contrast (all *t*s_(18)_ < 1.72, *ns*). The task was similar to study 1 except that the 16 blocks now consisted of 20 trials each (= 320 trials in total) and inter-trial interval was changed to 1000 ms.

#### Procedure

Participants were tested between 8:00 a.m. and 8:00 p.m. (median of testing time was 12:45 p.m.). All participants were asked not to consume food at least 3 h before the experiment. After participants had signed informed consent, half of the participants filled out the FCQ-S and subsequently consumed a bowl of cream kefir (Desira® calories and nutrients per serving (250 g): 360 kcal, 5.8 g protein, 42.3 g carbohydrates, 18.8 g fat) the taste of which is comparable to sweet fruit yoghurt. Before the AST, all participants filled out the FCQ-S (i.e., half of participants did this for the first time and half of participants did this for the second time). After participants had performed the AST, they immediately filled out the FCQ-S again and completed the other questionnaires. Finally, participants' height and weight was measured.

#### Data analyses

***Current food craving***. Groups (i.e., hungry vs. satiated) were compared for age, BMI, and food deprivation with independent *t*-tests and for dieting and smoking status with χ^2^-tests. Baseline levels of food craving (i.e., FCQ-S scores immediately before the AST in the hungry group and FCQ-S scores before food intake in the satiated group) were compared with an independent *t*-test. In the satiated group, changes in FCQ-S scores before and after food intake were tested with a paired *t*-test. Group differences and changes before and after the AST were tested with a 2 (between-subject factor *group*: hungry vs. satiated) × 2 (within-subject factor *time*: before vs. after the task) ANOVA for repeated measures.

***Correlates of current food craving***. Relationships of food deprivation, BMI, dieting success, and impulsivity with state food cravings (FCQ-S before the task, FCQ-S after the task, and the difference FCQ-S after the task minus FCQ-S before the task) were examined with linear regression analyses. For each of those variables, a regression model was calculated including the respective variable as well as group and an interaction term of both as predictors of state food cravings.

***Task performance***. Calculation of task performance was similar to study 1 and correlations between task performance indices are shown in Table 2. A 2 (within-subject factor *target type*: food vs. object) × 2 (within-subject factor *block type*: shift vs. non-shift) × 2 (between-subject factor *group*: hungry vs. satiated) ANOVA for repeated measures was calculated for each dependent variable (reaction time, omission, and commission errors). *Post-hoc t*-tests were calculated in case of significant interactions.

***Correlates of task performance***. Relationships of food deprivation, state food craving (i.e., difference of FCQ-S scores after the task minus FCQ-S scores before the task), BMI, dieting success, and impulsivity with task performance were examined with linear regression analyses. For each of those variables, a regression model was calculated including the respective variable as well as group (i.e., hungry vs. satiated) and an interaction term of both as predictors of task performance.

## Results

### Current food craving

**Figure 4:**
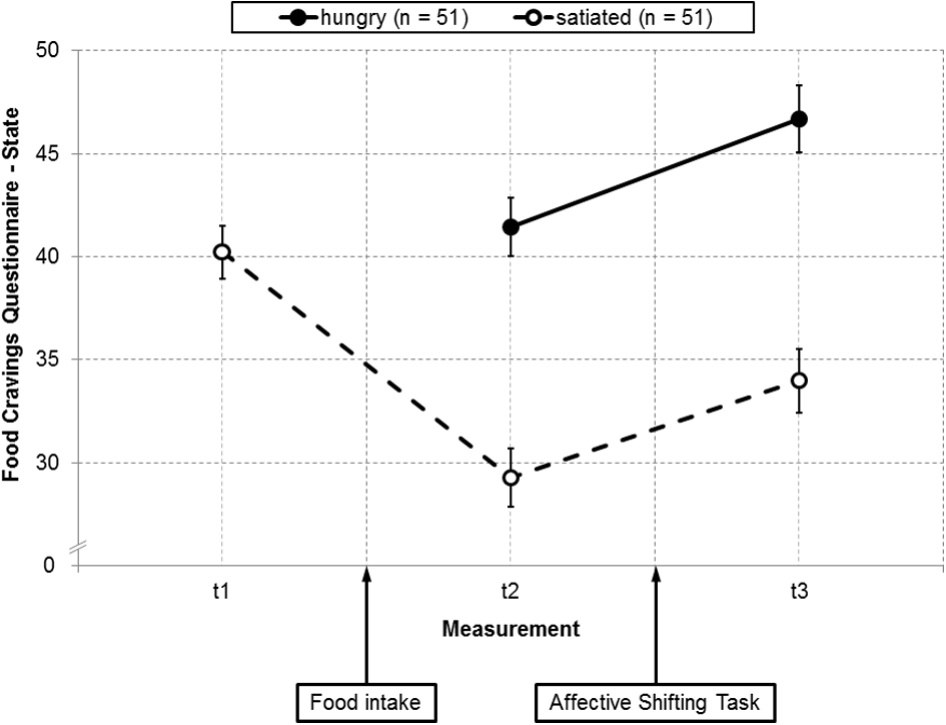
**Scores on the *Food Cravings Questionnaire—State* as a function of group before and after the *Affective Shifting Task* in study 2**.

Groups did not differ in age, BMI, food deprivation [all *t*s_(100)_ < 1.09, *ns*] or the proportion of current dieters and smokers [both χ^2^s_(1)_ < 0.55, *ns*]. They also did not differ in baseline craving levels, that is, when the FCQ-S was completed for the first time after arrival in the laboratory [i.e., FCQ-S scores before food intake in the satiated group vs. FCQ-S scores before the task in the hungry group; *t*_(100)_ = 0.65, *ns*]. In the satiated group, craving was reduced after food intake (*M* = 29.28, *SD* = 10.11) as compared to before [*M* = 40.22, *SD* = 9.16, *t*_(50)_ = 10.19, *p* < 0.001]. The ANOVA for craving before and after the task revealed significant main effects for group [*F*_(1, 100)_ = 37.31, *p* < 0.001, η^2^_*p*_ = 0.27] and time [*F*_(1, 100)_ = 65.87, *p* < 0.001, η^2^_*p*_ = 0.40] such that the hungry group reported higher craving (*M* = 44.07, *SD* = 10.46) than the satiated group (*M* = 31.63, *SD* = 10.11) and craving was higher after the task (*M* = 40.33, *SD* = 12.94) compared to before (*M* = 35.36, *SD* = 11.80, Figure 4). The interaction *group* × *time* was not significant [*F*_(1, 100)_ = 0.67, *ns*]^4^.

### Correlates of current food craving

**Figure 5:**
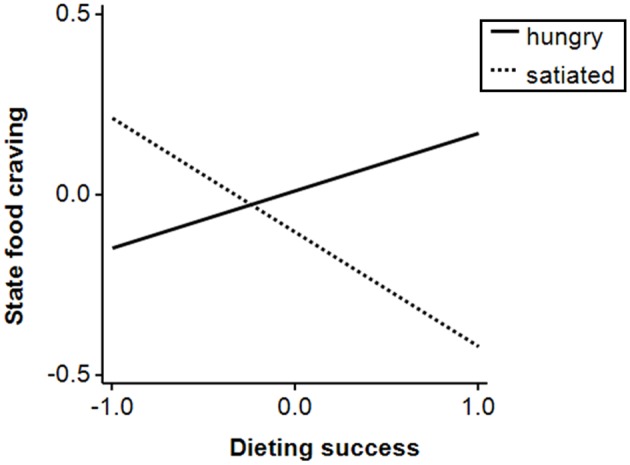
**State food craving (scores on the *Food Cravings Questionnaire—State* after the task minus scores before the task) as a function of group and dieting success in study 2**. All variables are *z*-standardized.

Similar to the findings above, group predicted food craving before and after the task such that the hungry group reported higher food craving (Table 4). BMI negatively predicted food craving before and after the task. Finally, the interaction *group* × *dieting success* significantly predicted the difference between food craving before and after the task: dieting success negatively predicted increases in food craving in the satiated group (β = −0.32, *p* < 0.05), but not in the hungry group (β = 0.16, *ns*, Figure 5).

**Table 4:** **Prediction of state food craving as a function of food deprivation, BMI, dieting success, impulsivity, and group (study 2)**.

	**FCQ-S before task**		**FCQ-S after task**		**FCQ-S difference**	
	**β**	***p***	**β**	***p***	**β**	***p***
Food deprivation						
Group	**−0.53**	**<0.001**	**−0.50**	**<0.001**	−0.05	*ns*
Food deprivation	0.15	*ns*	0.15	*ns*	0.02	*ns*
Group × food deprivation	−0.14	*ns*	−0.08	*ns*	0.10	*ns*
BMI						
Group	**−0.51**	**<0.001**	**−0.48**	**<0.001**	−0.04	*ns*
BMI	**−0.18**	**<0.05**	**−0.19**	**<0.05**	−0.06	*ns*
Group × BMI	0.07	*ns*	0.07	*ns*	0.03	*ns*
PSRS						
Group	**−0.50**	**<0.001**	**−0.48**	**<0.001**	−0.06	*ns*
PSRS	0.11	*ns*	0.06	*ns*	−0.08	*ns*
Group × PSRS	−0.06	*ns*	−0.16	*ns*	**−0.23**	**<0.05**
BIS−15						
Group	**−0.52**	**<0.001**	**−0.49**	**<0.001**	−0.04	*ns*
BIS−15	−0.01	*ns*	−0.06	*ns*	−0.11	*ns*
Group × BIS−15	0.01	*ns*	−0.02	*ns*	−0.07	*ns*

*FCQ-S, Food Cravings Questionnaire—State; BMI, Body mass index; PSRS, Perceived Self-Regulatory Success in Dieting Scale; BIS-15, Barratt Impulsiveness Scale—Short form*.

### Task performance

#### Reaction times

**Figure 6:**
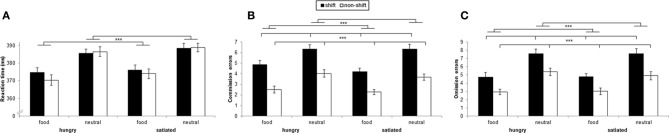
**Task performance as a function of target type, block type, and group in study 2**. Displayed is **(A)** reaction time in go-trials in ms, **(B)** number of commission, and **(C)** omission errors. Error bars indicate standard errors of the mean. ^***^p < 0.001.

There was a main effect for target type [*F*_(1, 100)_ = 116.15, *p* < 0.001, η^2^_*p*_ = 0.54] indicating that participants reacted faster in response to food targets (*M* = 373.72 ms, *SD* = 19.39) as compared to neutral targets (*M* = 386.85, *SD* = 17.26). No other effects were significant [all *F*s_(1,100)_ < 3.44, *ns*, η^2^_*p*_ < 0.04; Figure 6A].

#### Commission errors

There were main effects for target type [*F*_(1, 100)_ = 58.01, *p* < 0.001, η^2^_*p*_ = 0.37] and block type [*F*_(1, 100)_ = 130.66, *p* < 0.001, η^2^_*p*_ = 0.57] indicating that participants committed more errors in neutral blocks (*M* = 10.20 errors, *SD* = 4.32) compared to food blocks (*M* = 6.92 errors, *SD* = 4.16) and in shift blocks (*M* = 10.87 errors, *SD* = 4.87) compared to non-shift blocks (*M* = 6.25 errors, *SD* = 3.34). No other effects were significant [all *F*s_(1,100)_ < 1.28, *ns*, η^2^_*p*_ < 0.02; Figure 6B].

#### Omission errors

There were main effects for target type [*F*_(1, 100)_ = 94.83, *p* < 0.001, η^2^_*p*_ = 0.49] and block type [*F*_(1, 100)_ = 68.46, *p* < 0.001, η^2^_*p*_ = 0.41] indicating that participants omitted more targets in neutral blocks (*M* = 12.70 errors, *SD* = 6.86) compared to food blocks (*M* = 7.75 errors, *SD* = 5.39) and in shift blocks (*M* = 12.32 errors, *SD* = 6.93) compared to non-shift blocks (*M* = 8.12 errors, *SD* = 5.30). No other effects were significant [all *F*s_(1,100)_ < 2.69, *ns*, η^2^_*p*_ < 0.03; Figure 6C].

### Correlates of task performance

Group, food deprivation, and state food craving did not predict task performance whatsoever (Table 5). The interaction *group* × *BMI* predicted omission errors in food blocks: BMI positively predicted omission errors in food blocks in the hungry group (β = 0.34, *p* < 0.05), but not in the satiated group (β = −0.16, *ns*).

**Table 5:** **Prediction of task performance as a function of food deprivation, state food craving, BMI, dieting success, impulsivity, and group (study 2)**.

	**Reaction times**						**Commission errors**						**Omission errors**					
	**Food**		**Neutral**		**Food-neutral**		**Food**		**Neutral**		**Food-neutral**		**Food**		**Neutral**		**Food-neutral**	
	**β**	***p***	**β**	***p***	**β**	***p***	**β**	***p***	**β**	***p***	**β**	***p***	**β**	***p***	**β**	***p***	**β**	***p***
Food deprivation																		
Group	0.07	*ns*	0.05	*ns*	0.03	*ns*	−0.11	*ns*	−0.04	*ns*	−0.07	*ns*	0.01	*ns*	−0.03	*ns*	0.05	*ns*
Food deprivation	−0.05	*ns*	−0.09	*ns*	0.04	*ns*	0.04	*ns*	0.02	*ns*	0.02	*ns*	0.02	*ns*	−0.04	*ns*	0.08	*ns*
Group × food deprivation	−0.18	*ns*	−0.10	*ns*	−0.15	*ns*	−0.10	*ns*	0.10	*ns*	−0.19	*ns*	−0.04	*ns*	−0.07	*ns*	0.05	*ns*
FCQ−S difference^*^																		
Group	0.06	*ns*	0.05	*ns*	0.03	*ns*	−0.11	*ns*	−0.03	*ns*	−0.07	*ns*	0.01	*ns*	−0.03	*ns*	0.05	*ns*
FCQ−S difference	−0.05	*ns*	0.02	*ns*	−0.11	*ns*	0.02	*ns*	0.10	*ns*	−0.08	*ns*	0.00	*ns*	0.14	*ns*	−0.19	*ns*
Group × FCQ−S difference	0.05	*ns*	0.04	*ns*	0.03	*ns*	0.10	*ns*	−0.02	*ns*	0.12	*ns*	−0.02	*ns*	0.07	*ns*	−0.11	*ns*
BMI																		
Group	0.06	*ns*	0.04	*ns*	0.04	*ns*	−0.11	*ns*	−0.04	*ns*	−0.07	*ns*	0.01	*ns*	−0.05	*ns*	0.07	*ns*
BMI	0.10	*ns*	0.13	*ns*	−0.03	*ns*	0.06	*ns*	−0.01	*ns*	0.07	*ns*	0.09	*ns*	0.19	*ns*	−0.17	*ns*
Group × BMI	−0.13	*ns*	−0.05	*ns*	−0.13	*ns*	−0.10	*ns*	−0.05	*ns*	−0.04	*ns*	**−0.25**	**<0.05**	−0.10	*ns*	−0.13	*ns*
PSRS																		
Group	0.05	*ns*	0.01	*ns*	0.07	*ns*	−0.12	*ns*	−0.07	*ns*	−0.05	*ns*	−0.02	*ns*	−0.08	*ns*	0.09	*ns*
PSRS	−0.07	*ns*	**−0.22**	**<0.05**	**0.20**	**<0.05**	−0.07	*ns*	−0.19	*ns*	0.11	*ns*	−0.14	*ns*	**−0.25**	**<0.05**	0.19	*ns*
Group × PSRS	**0.21**	**<0.05**	0.13	*ns*	0.16	*ns*	**0.20**	**<0.05**	0.01	*ns*	0.18	*ns*	**0.36**	**<0.001**	0.06	*ns*	**0.30**	**<0.01**
BIS−15																		
Group	0.06	*ns*	0.05	*ns*	0.04	*ns*	−0.12	*ns*	−0.05	*ns*	−0.06	*ns*	0.01	*ns*	−0.04	*ns*	0.06	*ns*
BIS−15	0.02	*ns*	0.04	*ns*	−0.03	*ns*	0.12	*ns*	**0.27**	**<0.01**	−0.15	*ns*	0.11	*ns*	0.08	*ns*	0.00	*ns*
Group × BIS−15	−0.01	*ns*	0.14	*ns*	**−0.21**	**<0.05**	0.02	*ns*	**0.24**	**<0.05**	**−0.22**	**<0.05**	0.07	*ns*	0.06	*ns*	−0.01	*ns*

*FCQ-S, Food Cravings Questionnaire—State; BMI, Body mass index; PSRS, Perceived Self-Regulatory Success in Dieting Scale; BIS-15, Barratt Impulsiveness Scale—Short form*.

* *Absolute values (i.e., food craving before and after the task) also were uncorrelated to task performance*.

**Figure 7:**
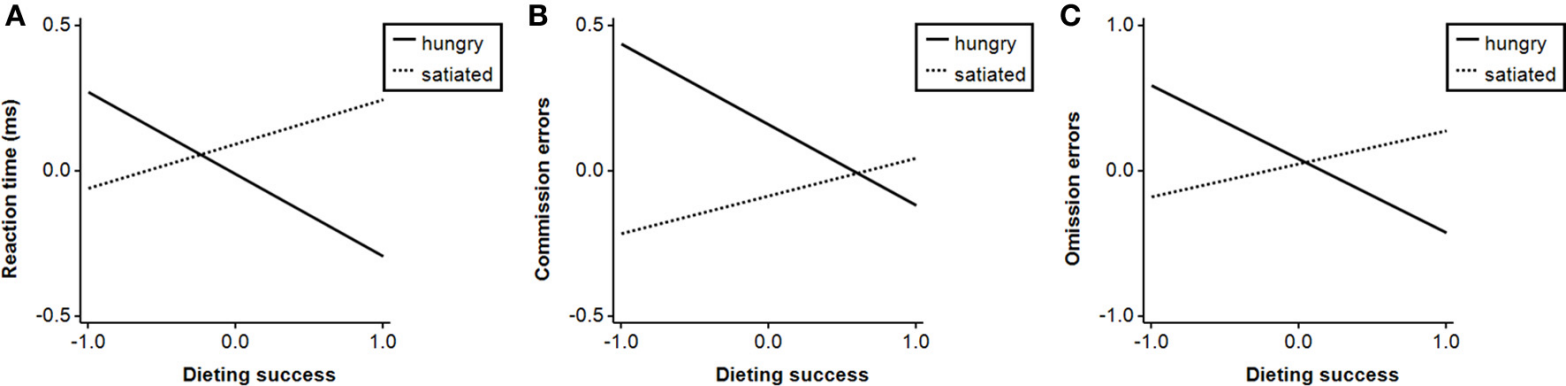
**Task performance in blocks with food targets as a function of group and dieting success in study 2: (A) reaction time in go-trials in ms, (B) number of commission, and (C) omission errors**. All variables are *z*-standardized.

Dieting success predicted faster reaction times and fewer omission errors in neutral blocks and slower reaction times in food as compared to neutral blocks (Table 5). There were further significant interactions of *group* × *dieting success* when predicting task performance in food blocks (Table 5): dieting success was associated with faster reaction times (β = −0.28, *p* < 0.05), fewer commission errors (β = −0.28, *p* < 0.05) and omission errors (β = −0.51, *p* < 0.001) in hungry participants, but not in satiated participants (all βs < 0.24, *ns*; Figure 7). In satiated participants, dieting success was associated with more omission errors in food relative to neutral blocks (β = 0.49, *p* < 0.001), which was not found in hungry participants (β = 0.22, *ns*).

In neutral blocks, self-reported impulsivity was associated with more commission errors (Table 5). There were further significant interactions of *group* × *impulsivity* when predicting commission errors: impulsivity was associated with more commission errors in neutral blocks (β = 0.50, *p* < 0.001) and with fewer commission errors in food as compared to neutral blocks (β = −0.36, *p* < 0.01) in satiated participants, but not in hungry participants (βs < 0.08, *ns*). Although there was also a significant interaction when predicting reaction times, beta-weights in both groups were not significant (βs < 0.24, *ns*).

## Conclusion of study 2

### Current food craving

In study 2, half of participants consumed food prior to performing the task, which successfully reduced their current food craving throughout the task as compared to the hungry group. Yet, state food craving was increased after the task as compared to before in both groups.

### Correlates of current food craving

Higher state food craving both before and after the task was associated with lower BMI and it remains speculative if this can be explained by physiological mechanisms or a reporting bias such as social desirability. Increases of state food craving during the task were predicted by lower dieting success in satiated, but not in hungry participants. This finding may represent a preload-disinhibition effect (Herman and Mack, 1975). Specifically, rather unsuccessful dieters have been found to increase their food intake after consumption of a high-calorie preload (Stroebe, 2008), the mechanisms of which may be similarly reflected by higher food-cue elicited craving with decreasing dieting success in the current study.

### Task performance

Replicating findings of study 1, study 2 showed that participants reacted faster in response to food than neutral targets and omitted fewer food than neutral targets. Contrary to study 1, participants also committed fewer errors in food blocks. Thus, results replicate findings showing decreased behavioral inhibition in neutral blocks (i.e., more impulsive reactions to food distractors) in the AST (Loeber et al., 2012, 2013). Differences in all three task performance indices between food and neutral blocks were unaffected by food intake, that is, did not differ between hungry and satiated individuals.

### Correlates of task performance

Similar to study 1, food deprivation and state food craving were unrelated to task performance. Hungry participants omitted more food targets with higher BMI and lower dieting success, respectively. Associations could not be found in satiated participants and, in fact, relationships seemed to be reversed as satiated participants omitted fewer food than neutral targets with lower dieting success. Higher dieting success also was associated with faster reaction times and fewer commission errors in hungry, but not in satiated participants. Finally, self-reported impulsivity was related to more commission errors in neutral blocks in the satiated group only.

To summarize, results show that food-cue affected behavioral inhibition is unrelated to current hunger or craving, that is, that differences in task performance between food and neutral stimuli cannot be explained by those variables. However, hunger and satiation does indeed influence the relationship between task performance with dieting success and self-reported impulsivity. Specifically, individuals with higher dieting success outperformed those with lower dieting success in food blocks as they reacted faster, omitted fewer targets, and committed fewer errors, but only when hungry. Moreover, higher self-reported impulsivity was related to decreased behavioral inhibition in neutral blocks, but only when satiated.

## Discussion

In the current studies, we investigated the influence of food-cues on behavioral inhibition using a pictorial AST. In study 1, participants showed accelerated reaction times in response to food targets as compared to neutral targets. They also omitted less food than neutral targets. Those findings could be replicated in study 2 and are in line with previous studies which used lexical food-related ASTs (Mobbs et al., 2008, 2011; Loeber et al., 2012). Faster reaction times in response to food targets may reflect an approach tendency toward positive stimuli. For example, faster reaction times in response to happy faces as compared to sad faces could be found in a comparable emotional go/no-go task (Schulz et al., 2007). In both of the present studies, those task performance indices were unrelated to current food deprivation and state food craving and, in study 2, were not influenced by food intake. Thus, it appears that faster reactions and fewer attentional lapses in response to food pictures may be due to the fact that they are generally salient stimuli because of their motivational relevance for survival and, thus, are more salient than other stimuli even in the absence of hunger. Contrarily, the number of omission errors in food blocks was reduced with higher self-reported hunger in a recent study by Loeber et al. (2013). In that study, however, omission errors were unrelated to current blood glucose levels which suggests that—in line with the current findings—a physiological mechanism due to energy deficit can unlikely account for those behavioral responses.

Another explanation for differences in reaction times and omission errors between food and neutral blocks could simply be a category size effect (Landauer and Freedman, 1968). Food pictures may be recognized faster than neutral pictures as the category of food is smaller than the broader category of neutral objects. Loeber et al. (2013) tried to solve this problem by using clothing words only as control category. Unfortunately, reaction times were not reported in that study. To conclude, a possible category size effect underlying the differences in those task performance indices cannot be fully ruled out.

Results of commission errors differed between study 1 and 2. In study 1, commission errors did not differ between target types. In study 2, participants committed fewer errors in food than neutral blocks. Results are probably different because we increased the total number of trials in study 2 and, subsequently, participants committed more errors. The increased number of commission errors in neutral blocks (i.e., false responses to food distractors) replicates findings of the lexical AST by Loeber et al. (2012, 2013). Thus, it appears that food-cues trigger automatic action tendencies which may be due to incentive salience. Commission errors were not affected by food deprivation, state food craving, or food intake in the present studies, but were associated with self-reported hunger and unrelated to blood glucose levels in the study by Loeber et al. (2013). Thus, it appears that food-cue affected response inhibition—similar to reaction times and omission errors—is also not influenced by physiological processes due to current nutritional status. However, it may depend on subjectively perceived hunger (at least when it is assessed by the *Grand Hunger Scales*, cf. Loeber et al., 2013).

Commission errors were associated with BMI and dieting success. In study 1, results indicated a more pronounced disinhibition in food relative to neutral blocks with lower dieting success and higher BMI. Those findings could partially be replicated in hungry participants in study 2 such that lower dieting success was associated with increased disinhibition in food blocks. In a recent study by Houben et al. (2012b), unsuccessful dieters also were less inhibited in a food-related stop-signal task after food exposure while successful dieters were not affected. Our results corroborate these findings in that both successful and unsuccessful dieters may be equally tempted by palatable food-cues, but only in successful dieters a control mechanism may be activated by such cues (Stroebe et al., 2013; Van Koningsbruggen et al., 2013), which may be reflected in increased inhibitory control in food blocks in the current studies.

While food intake did not influence task performance in general, it did modify task performance as a function of dieting success. That is, associations between task performance and dieting success, which were found in hungry participants, were vanished or even reversed in satiated participants. This finding may in part be explained by a preload-disinhibition effect (Herman and Mack, 1975). Just as unsuccessful dieters are known to increase food intake after consumption of a preload (Ruderman, 1986; Heatherton et al., 1988), low dieting success was associated with stronger increases in food craving during the task after food intake. However, current food craving was unrelated to task performance, so changes in task performance as a result of food intake and dieting success may not be explained by changes in current food craving. To conclude, although associations between food-related response inhibition and dieting success are not clear-cut, results do show that task performance is not independent of dieting success and that relationships can be modified by hunger and satiation.

Another interesting finding of the present studies was that self-reported impulsivity was unrelated to behavioral inhibition in hungry participants, but relationships could be observed in satiated participants. That is, satiated participants committed more errors in neutral blocks with increasing self-reported impulsivity. This finding has important implications for future research. For instance, a recent meta-analysis showed that there is a positive relationship between impulsivity and substance-related attentional bias (Coskunpinar and Cyders, 2013). While this relationship was not moderated by the type of substance (e.g., food or drugs of abuse), there was a stronger relationship between attentional bias and behavioral impulsivity (as assessed with behavioral tasks) than trait impulsivity (as assessed with questionnaires). The current findings imply that current nutritional status may be an important moderator of the relationship between trait impulsivity and behavioral reactions to substance-related cues. Specifically, while most studies usually instruct participants to be moderately food deprived, stronger relationships between trait impulsivity and food-related measures such as attentional bias may be found than previously reported when satiated participants are investigated. This may in part be explained by the fact that the influence of situational factors, such as mechanisms of homeostatic hunger, are reduced thereby increasing the influence of trait-related measures such as self-reported impulsivity (Lowe and Butryn, 2007; Lowe, 2009; Coskunpinar and Cyders, 2013). As a result, food-related attentional bias or food-cue induced behavioral disinhibition may be revealed as an important mediator between trait impulsivity and overeating (Hou et al., 2011; Meule, 2013).

## Future directions

It appears that an effect of food-cues on behavioral inhibition can only be observed when those cues are actually used as targets and distractors, respectively (Mobbs et al., 2008, 2011; Houben et al., 2012b; Loeber et al., 2012, 2013; Nederkoorn et al., 2012; Meule et al., 2014), but not when neutral stimuli, e.g., letters, are used which are just accompanied by food-cues (Meule et al., 2011a, 2012c) or food-exposure (Nederkoorn et al., 2004). Unlike previous studies, which used a lexical food-related AST, we used food pictures which arguably have higher external validity compared with food words and, as a result, produce larger effects in cognitive tasks (Brooks et al., 2011). However, a limitation of the current study is that we did not use another salient non-food category (cf. Nederkoorn et al., 2012) or a food-related control condition, for example, low-calorie food items (cf. Meule and Kübler, in revision). As a result, the influence of possible category size effects or similar cannot be excluded and future studies are needed that take this into account.

With regard to clinical implications, recent studies show that impulsivity or behavioral disinhibition is modifiable (Guerrieri et al., 2009, 2012) and that inhibition training reduces subsequent food intake (Houben, 2011; Houben and Jansen, 2011). For instance, when relevant stimuli (e.g., pictures of chocolate) were paired with a stopping response, a decrease of snack food intake in individuals with low inhibitory control (Houben, 2011) and a decreased chocolate consumption in chocolate cravers (Houben and Jansen, 2011) could be observed. Furthermore, consistently pairing palatable food-cues with stop signals has also been found to reduce consumption of those foods in chronic dieters (Veling et al., 2011) and to influence food choice such that participants more often chose healthy snack foods and fewer sweets (Veling et al., 2013a; Van Koningsbruggen et al., in press). It is assumed that the mechanism of such interventions is a reduction of implicit affective reactions toward tempting stimuli rather than an increase in response inhibition (Houben et al., 2012a; Veling et al., 2013b). Unfortunately, neither of those studies assessed if those individuals whose food intake was altered after the training actually displayed behavioral disinhibition in response to the relevant food stimuli in the first place. Therefore, future studies may investigate if such training reduces food bias as assessed with the AST, that is, increases behavioral inhibition in food blocks, and if this behavioral change may also lead to a long-term decrease in unhealthy food consumption as well as more healthy food choices, particularly in unsuccessful dieters.

To conclude, the present studies demonstrated that pictorial, high-calorie food-cues affect motor response inhibition. Furthermore, task performance was differentially related to BMI, self-reported dieting success, and impulsivity as a function of hunger and satiation. Yet, we investigated a sample of healthy, young women with normal BMI. Future studies may extend the current findings and assess their clinical relevance by examining obese samples or individuals with eating disorders.

### Conflict of interest statement

The authors declare that the research was conducted in the absence of any commercial or financial relationships that could be construed as a potential conflict of interest.

## Footnotes

^1^Note that to be concise we did not review the comprehensive literature on other related tasks such as the Stroop, and instead focused only on studies using paradigms specifically assessing motor response inhibition without confounding other processes.

^2^The reported data were part of a study that also included other tasks and physiological recordings on three occasions, which are reported elsewhere (Meule et al., 2012b, d, 2014). The order of tasks was counterbalanced, that is, subjects performed the AST either on the first, second, or third session. Each session was performed on separate days with 1 week in between and none of the stimuli used in the AST were used in the other tasks. Thus, we would argue that it is unlikely that performing the other tasks adversely affected results obtained with the AST.

^3^Picture numbers in the *food.pics* database: 9, 10, 22, 32, 38, 82, 115, 145, 163, 167, 1001, 1003, 1008, 1011, 1012, 1020, 1025, 1031, 1032, 1034.

^4^Note that results for each FCQ-S subscale (i.e., *desire, reinforcement, relief, lack of control, hunger*) were similar to those for the total score. That is, scores on each subscale decreased after food intake in the satiated group, were lower before and after the task in the satiated as compared to the hungry group, and were increased in both groups after the task as compared to before (all *ps* < 0.01).
